# Multiple De Novo Cerebral Arteriovenous Malformations in a Patient with Alcoholic Liver Cirrhosis

**DOI:** 10.5334/jbsr.2822

**Published:** 2022-05-27

**Authors:** Taehoon Do, Seung-Jae Lee, Jungbin Lee

**Affiliations:** 1 Department of Neurology, Soonchunhyang University Bucheon Hospital, Bucheon, South Korea; 2 Department of Radiology, Soonchunhyang University Bucheon Hospital, Bucheon, South Korea

**Keywords:** arteriovenous malformation, intracranial, cerebral, liver cirrhosis, hepatic failure, multiple

## Abstract

Cerebral arteriovenous malformations (cAVMs) are traditionally considered congenital anomalies. The literature includes only two reported cases of de novo solitary cAVMs associated with liver cirrhosis (LC). Here, we report a unique case of multiple de novo cAVMs in a patient with alcoholic LC.

**Teaching point:** In patients with potential risks for de novo cAVM, including liver cirrhosis, the possibility of multiple de novo cAVMs should be considered.

## Introduction

Cerebral arteriovenous malformations (cAVMs) are traditionally considered as congenital anomalies. However, recent reports have suggested that they may be an acquired disease associated with specific conditions [1]. Herein, we report a unique case of multiple de novo cAVMs associated with liver cirrhosis (LC).

## Case report

**Figure 1:**
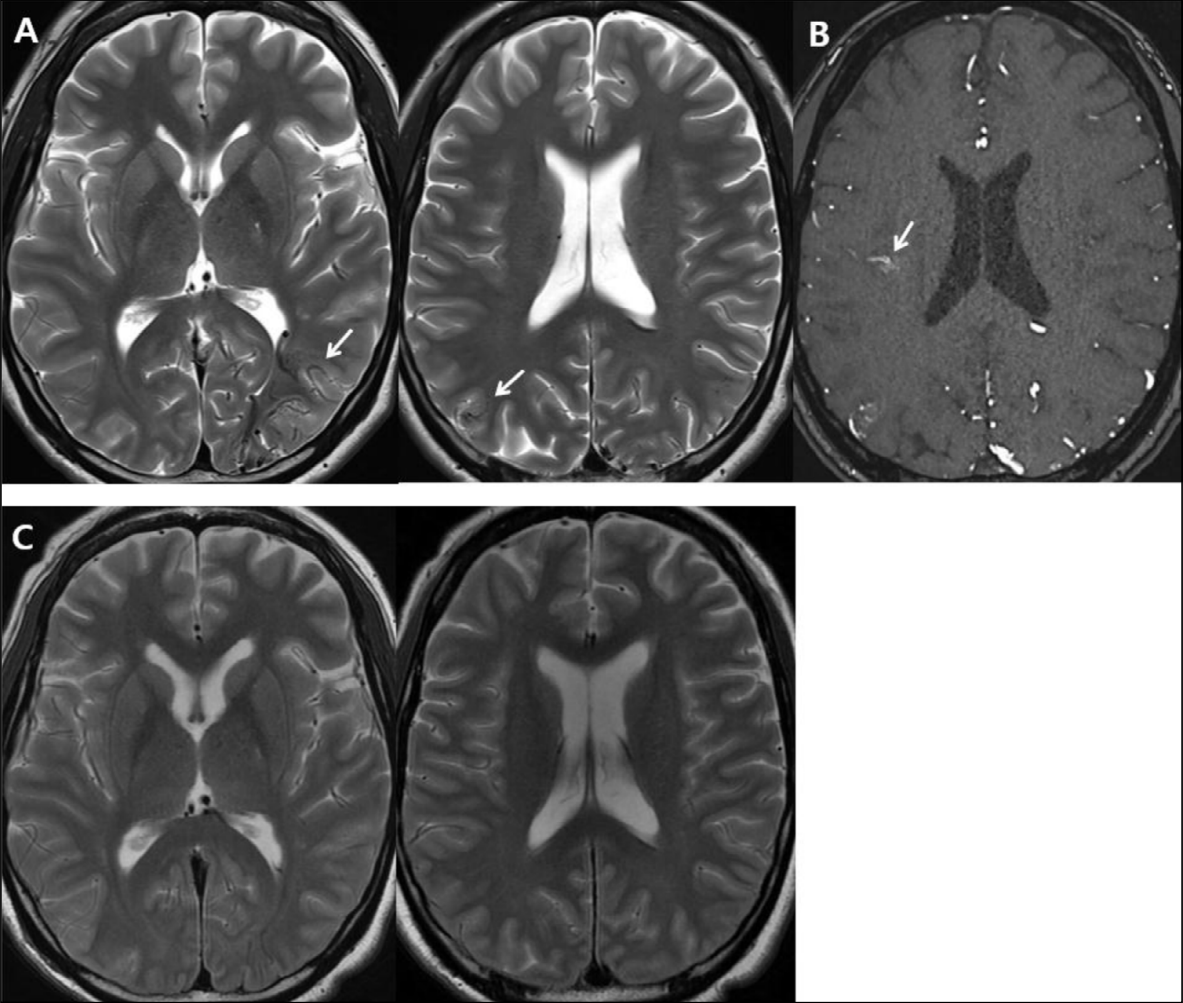
Magnetic resonance (MR) images: **A.** MR T2-weighted images revealed tortuous vascular structures with dark flow voids involving the left parieto-occipital and right parietal lobes (white arrows). **B.** Time-of-flight MR angiography source revealed a small cluster of vessels in the right insular lobe (white arrow). **C.** MR T2-weighted images acquired seven years earlier did not show any abnormal vascular structures.

**Figure 2:**
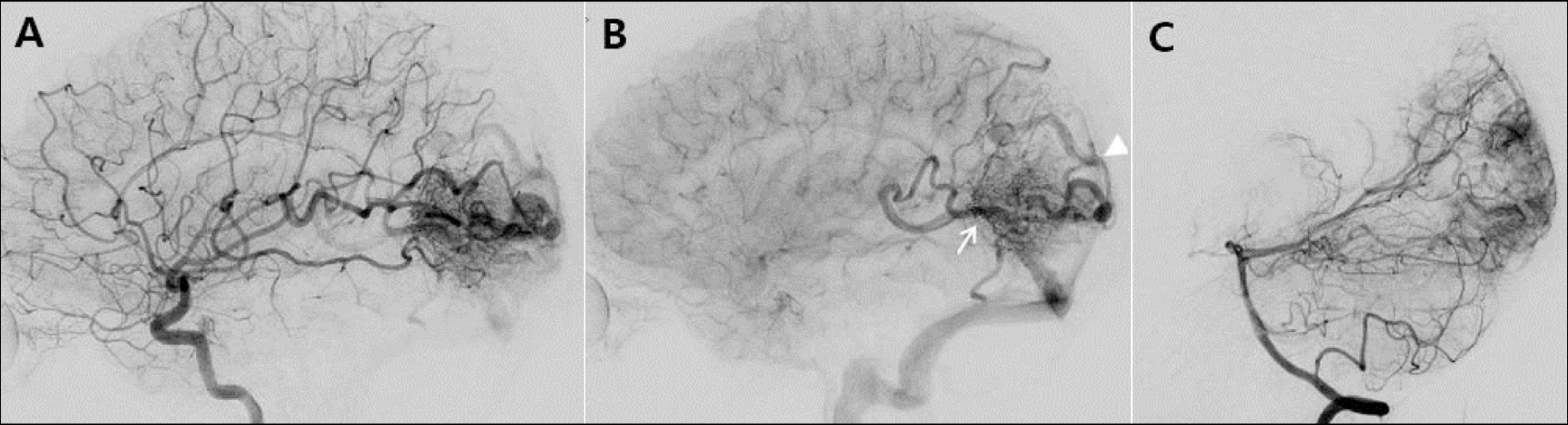
Digital subtraction angiography: **A** and **B.** Lateral left carotid angiogram revealed a left parieto-occipital arteriovenous malformation (AVM) with a nidus measuring 55 mm, which was drained via deep (vein of Galen and straight sinus: white arrow) and superficial (cortical veins and superior sagittal vein: white arrowhead) venous systems. **C.** Lateral vertebral angiogram demonstrated that the AVM was fed by the left posterior cerebral artery as well as the left middle cerebral artery.

**Figure 3:**
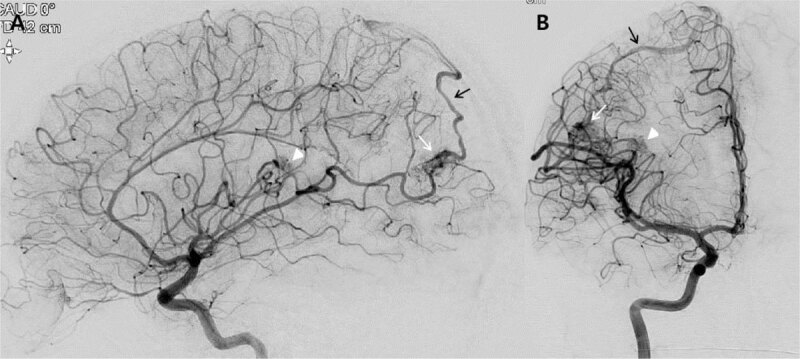
Digital subtraction angiography: **A** and **B.** Lateral and anteroposterior right carotid angiogram showed arteriovenous malformations (AVMs) in the right insular (white arrowhead) and parietal (white arrow) lobes, measuring 7 mm and 15 mm, respectively. The AVMs were drained though cortical veins to the superior sagittal sinus (black arrow).

A 33-year-old woman with a history of alcoholic liver cirrhosis presented with a focal seizure, which began with left sided paresthesia followed by tonic posturing. The electroencephalogram was normal. However, the brain magnetic resonance images (bMRIs) and digital subtraction angiography revealed multiple cAVMs (Figures 1A, 1B, 2 and 3), which were not found in the bMRIs acquired seven years earlier (Figure 1C).

## Discussion

We present a very rare case of cAVM that was confirmed to be an acquired form associated with LC. Only two cases of de novo cAVM associated with LC have been reported in the literature [2,3]. However, unlike the previously reported cases, our patient showed multiple cAVMs, which are known to only occur in 0.3%–4% of all cases of cAVMs, except in cases of hereditary autosomal disease [4]. To the best of our knowledge, this is the first case of multiple de novo cAVMs associated with LC.

Similarly, the AVMs associated with LC have also been reported in organs other than brain, such as the lower extremities [5], fingers [6], lungs [7], and ileocecum [8]. Among these, some cases showed spontaneous regression of AVMs after liver transplantation or recovery of hepatic function [6,7]. In addition, spontaneously resolving cAVMs after liver transplantation have also been reported [9].

The de novo appearance and regression of AVMs in relation to liver function may indicate a cause-and-effect relationship between hepatic failure and de novo AVM. The exact mechanism has not yet been clearly elucidated, but there is a persuasive explanation [1,9]. In cirrhotic livers, surviving hepatic cells release angiogenic factors such as vascular endothelial growth factor into the systemic circulation, promoting a proangiogenic condition.

In addition to LC, other diseases have been associated with de novo cAVMs, including preexisting intracranial vascular malformations, aneurysms, strokes, seizures, brain tumors, moyamoya disease, traumatic brain injury, and inflammatory lesions. Such pathological conditions may elicit a proangiogenic environment, acting as a ‘second hit’ for triggering cAVMs in individuals with genetic susceptibility [1].

## Conclusion

Here, we present a case of multiple de novo cAVMs associated with alcoholic LC. Therefore, in patients with potential risks for de novo cAVMs, including LC, the possibility of multiple de novo cAVMs should be considered, and the images should be carefully reviewed, even if previous brain images are normal.
